# Potential for increased photosynthetic performance and crop productivity in response to climate change: role of CBFs and gibberellic acid

**DOI:** 10.3389/fchem.2014.00018

**Published:** 2014-04-17

**Authors:** Norman P. A. Hüner, Keshav Dahal, Leonid V. Kurepin, Leonid Savitch, Jas Singh, Alexander G. Ivanov, Khalil Kane, Fathey Sarhan

**Affiliations:** ^1^Biology Department and the Biotron Centre for Experimental Climate Change Research, University of Western OntarioLondon, ON, Canada; ^2^Department of Biological Sciences, University of Toronto at ScarboroughScarborough, ON, Canada; ^3^Eastern Cereal and Oilseed Research Centre, Agriculture and Agri-Food CanadaOttawa, ON, Canada; ^4^Départment des Sciences biologiques, Université du Québec à MontréalMontréal, QC, Canada

**Keywords:** phenotypic plasticity, photosynthetic performance, crop productivity, CBFs, gibberellic acid, climate change

## Abstract

We propose that targeting the enhanced photosynthetic performance associated with the cold acclimation of winter cultivars of rye (*Secale cereale* L.), wheat (*Triticum aestivum* L.), and *Brassica napus* L. may provide a novel approach to improve crop productivity under abiotic as well as biotic stress conditions. In support of this hypothesis, we provide the physiological, biochemical, and molecular evidence that the dwarf phenotype induced by cold acclimation is coupled to significant enhancement in photosynthetic performance, resistance to photoinhibition, and a decreased dependence on photoprotection through non-photochemical quenching which result in enhanced biomass production and ultimately increased seed yield. These system-wide changes at the levels of phenotype, physiology, and biochemistry appear to be governed by the family of C-repeat/dehydration-responsive family of transcription factors (CBF/DREB1). We relate this phenomenon to the semi-dwarf, gibberellic acid insensitive (GAI), cereal varieties developed during the “green revolution” of the early 1960s and 1970s. We suggest that genetic manipulation of the family of C-repeat/dehydration-responsive element binding transcription factors (CBF/DREB1) may provide a novel approach for the maintenance and perhaps even the enhancement of plant productivity under conditions of sub-optimal growth conditions predicted for our future climate.

## Introduction

The increase in the yield of major food crops since the mid-1950s has been achieved mainly through genetic improvement and increased use of agricultural inputs such as fertilizers, pesticides, and water (Murchie et al., 2009). Zhu et al. (2010) have suggested that the yield of major food crops since the last decade is increasing slowly, which may indicate that yield increase due to improved agricultural practices has reached an upper theoretical limit. Thus, it appears that the further enhancement in crop yield can only be achieved by enhancing genetic yield potential, that is, the seed yield that a crop can achieve per unit ground area under optimum growth conditions without biotic and abiotic stresses. The maximum potential biomass and grain yield that a plant can produce is determined essentially by the following five yield variables: (a) the amount of incident solar radiation available over the growing season of a plant, (b) the light interception efficiency, that is, the efficiency of the photosynthetic pigments to intercept photosynthetic active radiation, (c) the energy conversion efficiency, that is, the ratio of the biomass energy produced over a given period to the radiative energy intercepted by the canopy over the same period, (d) the translocation of photosynthates to sinks, as determined by sink strength, and (e) the partitioning efficiency, that is, the amount of total biomass energy partitioned into seed production per unit ground area, also known as harvest index (HI) (Loomis and Amthor, 1999; Long et al., 2006; Zhu et al., 2010).

Since energy partitioning efficiency and light interception efficiency have approached the theoretical upper limit (Zhu et al., 2010), further increase in yield potential can only be achieved by an increase in the energy conversion efficiency into biomass. Since plant dry matter consists of about 40% carbon by weight, an increase in total biomass production can be achieved through enhanced photosynthetic carbon assimilation (Murchie et al., 2009). Although photosynthesis is the ultimate basis for the conversion of light energy into biomass and seed yield to date, improving photosynthetic carbon assimilation has played only a minor role in enhancing energy conversion to biomass and seed yield (Long et al., 2006; Zhu et al., 2010).

This is, in part, due to the important photoprotective mechanisms that have evolved in all photoautotrophs to protect the photosynthetic apparatus from irradiance that is in excess of that which can be utilized for either reductive CO_2_ assimilation as well as reductive N and S assimilation (Adams III and Demmig-Adams, 1993; Demmig-Adams and Adams III, 1996; Horton et al., 1996; Demmig-Adams et al., 1999; Niyogi, 1999; Horton, 2000, 2012; Horton and Ruban, 2005). Excess light is not only necessarily due to an increase in absolute actinic irradiance but is also generated with no change in absolute irradiance when coupled with either low temperature (Krause, 1994; Hüner et al., 1998; Ensminger et al., 2006; Farage et al., 2006; Takahashi and Murata, 2008; Hüner and Grodzinski, 2011) or other abiotic and biotic environmental stresses (Murata et al., 2007; Takahashi and Murata, 2008) by decreasing the photochemical efficiency of PSII. Consequently, the induction of photoprotective mechanisms to dissipate excess absorbed energy represents an important mechanism to balance the flux of energy absorbed and transformed into reducing power with the flux of energy utilized through the consumption of photosynthetically generated electrons by C, N, and S metabolism (Anderson et al., 1995; Hüner et al., 1998, 2013; Öquist and Hüner, 2003; Eberhard et al., 2008; Murchie et al., 2009; Hüner et al., 2012). Although the induction of photoprotective mechanisms reduces the efficiency of CO_2_ assimilation and decreases biomass production, these photoprotective mechanisms are essential for plant survival against myriad environmental stresses (Kulheim et al., 2002; Sane et al., 2012). Even though inhibition of these photoprotective mechanisms would theoretically increase energy conversion efficiency in the very short-term, this is simply not an option for long-term survival of plants exposed to environmental conditions that fluctuate on an hourly, daily, and annual basis. Photoprotection from excess irradiance is essential for maximizing plant fitness and survival (Kulheim et al., 2002).

The increase in yield potential of major crops over the past 50 years occurred, by and large, as a consequence of improved partitioning efficiency and light interception efficiency of crop plants (Long et al., 2006; Murchie et al., 2009; Zhu et al., 2010). Increased partitioning efficiency has been accomplished through the release of semi-dwarf cultivars producing higher numbers of seeds per plant and an increase in HI. The new wheat varieties associated with the “green revolution” and introduced in the 20th century were generally shorter and exhibited an increased grain yield at the expense of straw biomass, that is, exhibited enhanced HI. The *gai* mutant alleles that control this shorter phenotype and increased HI encode mutant forms of the gibberellin response transcription factor, GAI (Peng et al., 1999). Increased light interception efficiency is a consequence of increased leaf area index associated with the development of the semi-dwarf cultivars with improved lodging resistance against the adverse weather conditions, such as rain, wind, and hail (Peng et al., 1999; Long et al., 2006; Murchie et al., 2009). Recently, it was reported that the rice cytokinin GATA transcription factor1 encoded by *Cga1* governs a dwarf phenotype as well as chloroplast development in rice (Hudson et al., 2013). Thus, altered *Cga1* expression appears to mimic the effects of altered gene expression in GA signaling associated with the elite “green revolution” wheat varieties.

Presently, we summarize experimental evidence that targeting the dwarf phenotype, and enhanced photosynthetic performance typically associated with the cold acclimated (CA) state of winter cereals and *Brassica napus* may represent a novel approach to improve crop yield and productivity. We show that the requirement for cold acclimation to enhance photosynthetic performance can be circumvented by overexpression of the C-repeat/dehydration responsive family of transcription factors. We suggest that this approach may provide important insights into potential molecular approaches focused on at least the maintenance or, perhaps, even the enhancement of plant productivity under sub-optimal growth conditions predicted to be associated with future climate change.

## Cold acclimation increases photosynthetic performance

**Figure 1:**
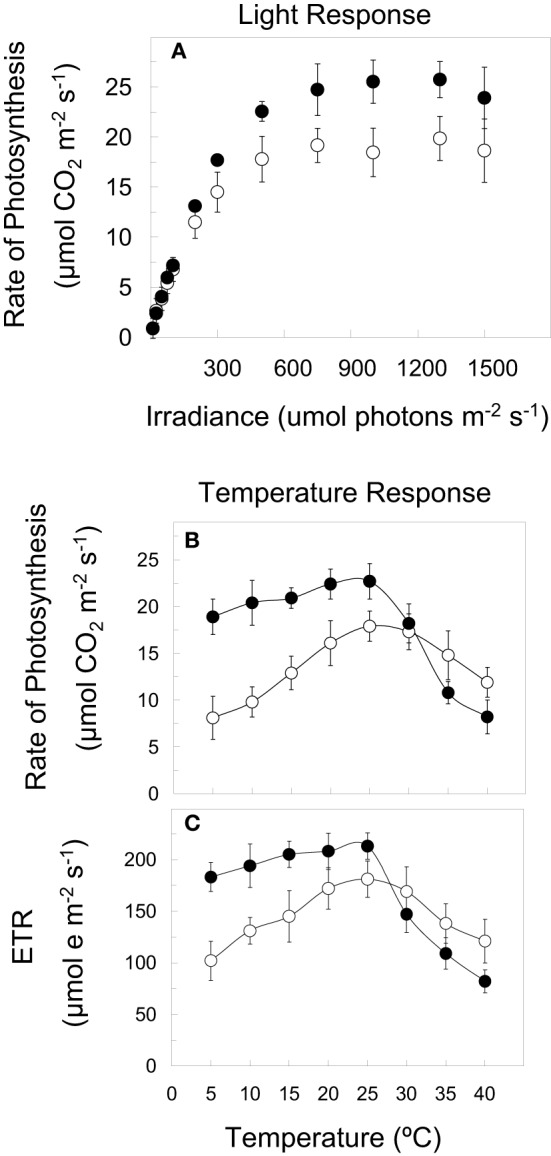
**Light and temperature response curves for winter rye (*Secale cereale* L. cv Musketeer)**. NA Musketeer (open symbols) was grown at 20°C and an irradiance of 250 μmol photons m^−2^ s^−1^ whereas CA Musketeer (closed symbols) was grown at 5°C and 250 μmol photons m^−2^ s^−1^ with a 16 h/8 h light/dark photoperiod. **(A)** Light response curves for CO_2_ assimilation were measured at the growth temperature under ambient CO_2_ conditions. Temperature response curves for CO_2_ assimilation **(B)** and ETR **(C)** represent light saturated rates under ambient CO_2_ conditions. All data are from Dahal et al. (2012a,b,c) with permission.

Previous studies have reported that CA winter cultivars of rye (Figure 1A), wheat, barley, Brassica, spinach, and *Arabidopsis thaliana* are characterized by an increased photosynthetic capacity relative to non-acclimated (NA) controls. Furthermore, the temperature response curves for both CO_2_ assimilation (Figure 1B) and photosynthetic electron transport (ETR) (Figure 1C) indicate that CA plants exhibit higher rates at all temperatures between 5 and 25°C relative to NA controls (Figures 1B,C) (Hüner et al., 1998; Öquist and Hüner, 2003; Dahal et al., 2012a,b). This has been accounted for by the up-regulation of carbon metabolism as a consequence of increased gene expression and activities of CO_2_-fixing enzyme, Rubisco (Hurry and Hüner, 1991; Hurry et al., 1994, 1995, 2000; Hüner et al., 1998; Strand et al., 1999; Öquist and Hüner, 2003; Dahal et al., 2012a,b) as well as enhanced activities of the cytosolic, sucrose biosynthetic enzymes, cFBPase, and SPS (Hurry et al., 1995a; Strand et al., 1999; Savitch et al., 2000a; Rapacz et al., 2008; Dahal et al., 2012a,b) in response to low growth temperature. In addition, winter cultivars of wheat (Savitch et al., 2000a; Leonardos et al., 2003) and *Arabidopsis thaliana* (Stitt and Hurry, 2002) combine an enhanced sink capacity with increased rates of sucrose export in response to cold acclimation. The major portion of this sucrose is stored as fructans in the crown tissue and leaf mesophyll cell vacuoles during cold acclimation of winter cereals (Pollock and Cairns, 1991; Savitch et al., 2000a). Consequently, cold acclimation of winter wheat, winter rape (Hurry et al., 1995a), and *Arabidopsis thaliana* (Stitt and Hurry, 2002) results in enhanced P_*i*_ cycling and increased capacity for RuBP regeneration. Concomitantly, cold acclimation of winter cereals suppresses photorespiration (Savitch et al., 2000b) and stimulates carbon export rates from source leaves (Leonardos et al., 2003). Consequently, the process of cold acclimation of winter cereals appears to co-ordinate system-wide readjustments in plant metabolism in feed-forward stimulation of photosynthetic CO_2_ fixation due to enhanced source-sink activities and increased capacity for carbon translocation (Hurry et al., 1995a; Strand et al., 1999; Stitt and Hurry, 2002; Leonardos et al., 2003). This is supported by a detailed, comparative metabolomics study of CA vs. NA *Arabidopsis thaliana* (Gray and Heath, 2005).

**Figure 2:**
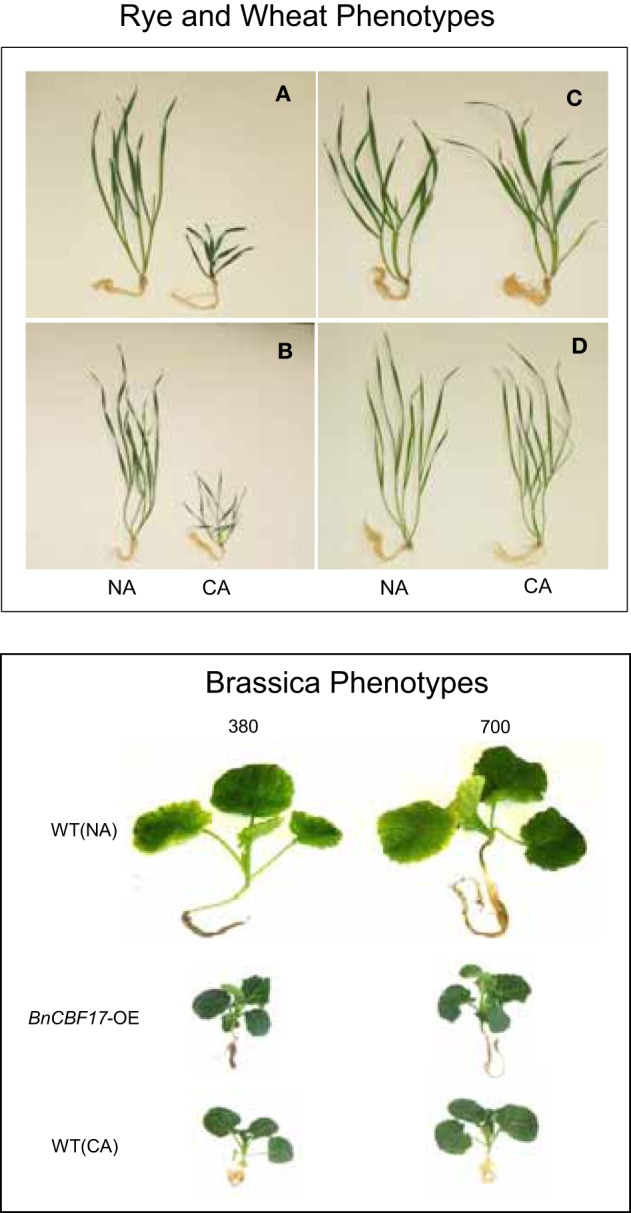
**The effect of cold acclimation on plant phenotype**. All plants were photographed at similar physiological states based on comparative growth kinetics. See Dahal et al. (2012a,b,c), for details. **(A)** NA and CA winter rye (*Secale cereale* L. cv Musketeer). **(B)** NA and CA Norstar winter wheat (*Triticum aestivum* L. cv Norstar). **(C)** NA and CA spring rye (*Secale cereale* L. cv SR4A). **(D)** NA and CA spring wheat (*Triticum aestivum* L. cv Katepwa). NA *Brassica napus* L. cv Westar WT and the *BnCBF*17 overexpressor (*BnCBF*17-OE) were grown at 20°C and an irradiance of 250 μmol photons m^−2^ s^−1^ with a 16 h/8 h light/dark photoperiod at either ambient (380 ppm CO_2_) or elevated CO_2_ (700 ppm CO_2_). CA *Brassica napus* L. cv Westar WT was grown at 5°C and an irradiance of 250 μmol photons m^−2^ s^−1^ with a 16 h/8 h light/dark photoperiod at either ambient (380 ppm CO_2_) or elevated CO_2_ (700 ppm CO_2_). Photographs are taken from Dahal et al. (2012a,b) with permission.

These adjustments at the physiological, biochemical, and molecular levels in response to cold acclimation are associated with coordinated changes in leaf anatomy and plant phenotype (Hüner, 1985; Gray et al., 1996, 1997; Strand et al., 1999; Dahal et al., 2012a,b) and induction of freezing tolerance in cold-tolerant species (Sarhan et al., 1997; Pocock et al., 2001; Thomashow, 2001; Savitch et al., 2005; Rapacz et al., 2008; Theocharis et al., 2012). With respect to phenotypic plasticity, cold acclimation of winter rye, winter wheat, and *Brassica napus* (Figure 2) as well as spinach and *Arabidopsis thaliana* generally results in a compact, dwarf phenotype (Hüner, 1985; Boese and Hüner, 1990; Strand et al., 1999; Savitch et al., 2005; Gorsuch et al., 2010a,b; Dahal et al., 2012a,b).

Previously, it has been assumed that the induction of the dwarf phenotype in overwintering herbaceous plants is regulated by low temperature (Levitt, 1980). However, we have shown that this is not the case. In fact, the phenotypic plasticity associated with cold acclimation of winter cultivars is regulated by the redox state of the chloroplast measured as excitation pressure (Gray et al., 1997; Hüner et al., 1998, 2012, 2013; Wilson et al., 2006; Kurepin et al., 2013). In addition, *WCS*19, a nuclear encoded gene originally associated with wheat freezing tolerance, has also been reported to be regulated by excitation pressure rather than by low temperature (Gray et al., 1997; NDong et al., 2001). Excitation pressure, estimated *in vivo* using Chl a fluorescence induction (Dietz et al., 1985; Hüner et al., 1998), is a quantitative measure of the proportion of closed PSII reaction centers due to an imbalance between energy absorbed vs. energy either utilized through metabolism and growth or dissipated as heat. Excitation pressure represents a chloroplast redox signal which emanates from the photosynthetic ETR chain and regulates not only the organization and composition of the photosynthetic apparatus but also phenotypic plasticity (Anderson et al., 1995; Hüner et al., 1998, 2003, 2012, 2013; Ensminger et al., 2006; Rosso et al., 2009; Kurepin et al., 2013).

The CA dwarf phenotype is also associated with altered leaf mesophyll cell ultrastructure and increased leaf thickness. The former is characterized by an increase in cytoplasmic volume combined with a decrease in vacuolar volume as estimated from cross sectional areas of transmission electron micrographs which are correlated with increased specific leaf weight relative to NA winter rye and *Arabidopsis thaliana* (Hüner et al., 1984; Strand et al., 1999). The latter can be accounted for by either increases in leaf mesophyll cell size (Hüner, 1985; Gorsuch et al., 2010a,b) or increases in palisade mesophyll layers (Boese and Hüner, 1990, 1992; Dahal et al., 2012a). These changes in leaf morphology are accompanied by an increase in the leaf protein content (Table 1) as well as an increase in the contents of sucrose and other structural carbohydrates (Guy et al., 1992; Hurry et al., 1995b; Strand et al., 1999, 2003; Savitch et al., 2000a,b; Gorsuch et al., 2010b). Consequently, the absolute shoot biomasses of the CA dwarf plants are comparable to the shoot biomasses of the NA plants with the larger, extended phenotype (Table 1). This indicates a significant increase in total energy per unit plant volume during the autumn cold acclimation period which is critical for winter survival and enhanced seed yield the following spring (Dahal et al., 2012b). Furthermore, the increased photosynthetic capacity exhibited by CA plants is, in part, due to the increased photosynthetic apparatus per unit leaf area (Dahal et al., 2012b). Functionally, this leads to an increased capacity to utilize absorbed light energy which keeps a greater proportion of PSII reaction centers open, that is, lowers excitation pressure (Hüner et al., 2003). This, in turn, results in a lower probability for light-dependent inhibition of photosynthesis due to photoinhibition (Krause, 1994, 1988; Osmond, 1994; Melis, 1999; Murata et al., 2007) under suboptimal growth conditions (Hurry and Hüner, 1992; Hüner et al., 1993; Gray et al., 1996, 1997). Although the co-ordination of such system-wide adjustments in photosynthesis, carbon metabolism, translocation, and phenotype appear to be correlated with the modulation of chloroplast excitation pressure, the precise molecular mechanism(s) that govern such complex, system-wide changes have yet to be elucidated. This remains a major challenge for future research.

**Table 1:** **Effects of cold acclimation on growth and photosynthesis**.

**Growth characteristic**	**Acclimation state**	**Musketeer**	**SR4A**	**Brassica WT**	***BnCBF*17**
Growth rate (day^−1^)	NA	0.225 ± 0.002	0.233 ± 0.003	nd	nd
	CA	0.074 ± 0.002	0.078 ± 0.002	nd	nd
A_*sat*_	NA	18.0 ± 2.8	22.2 ± 2.3	15.0 ± 3.1	23.1 ± 2.0
	CA	27.1 ± 2.5	15.1 ± 1.8	22.5 ± 2.5	nd
SLW (g m^−2^)	NA	37 ± 6	33 ± 5	27 ± 3	50 ± 6
	CA	90 ± 11	40 ± 4	63 ± 5	nd
Shoot dry mass (mg/plant)	NA	279 ± 12	337 ± 22	103 ± 6	122 ± 10
	CA	264 ± 32	349 ± 42	117 ± 12	nd
Leaf Protein (g m^−2^)	NA	3.67 ± 0.32	3.92 ± 0.44	3.65 ± 0.33	16.30 ± 1.50
	CA	10.6 ± 1.13	3.53 ± 0.33	17.5 ± 1.40	nd
WUE (A/gs)	NA	34 ± 5	29 ± 5	48 ± 6	63 ± 4
	CA	94 ± 12	32 ± 4	87 ± 13	nd

Winter rye (Secale cereale L. cv Musketeer); Spring rye (Secale cereale L. cv SR4A); WT, wild type; Brassica napus L. cv Westar; BnCBF17, CBF overexpressor of Brassica napus; SLW, specific leaf weight; A_sat_, light saturated rate of CO_2_ assimilation (μmol CO_2_ m^−2^ s^−1^); WUE, water use efficiency calculated as the ratio of light-saturated rates of CO_2_ assimilation (A) and stomatal conductance (gs). All data are from Dahal et al. (2012b, 2013b).

However, the ability to co-ordinate system-wide adjustments in photosynthetic performance in response to low growth temperature appears to be cultivar dependent. In contrast to winter cereals, spring cultivars do not exhibit this change in phenotype but rather maintain an elongated phenotype upon cold acclimation (Figure 2) with minimal changes in SLW and leaf protein content (Table 1) even though spring varieties are able to grow at low temperature (Dahal et al., 2012b, 2013b). Most likely this is due to the differential vernalization requirements for winter vs. spring varieties (Sung and Amasino, 2005; Oliver et al., 2009; Ko et al., 2010; Trevaskis, 2010). However, CA spring cereals exhibit a significant inhibition in photosynthetic capacity coupled with a much higher susceptibility to photoinhibition compared to CA winter cereals (Hurry and Hüner, 1991; Pocock et al., 2001; Dahal et al., 2012b).

## Cold acclimation minimizes dependence on NPQ for photoprotection

**Figure 3:**
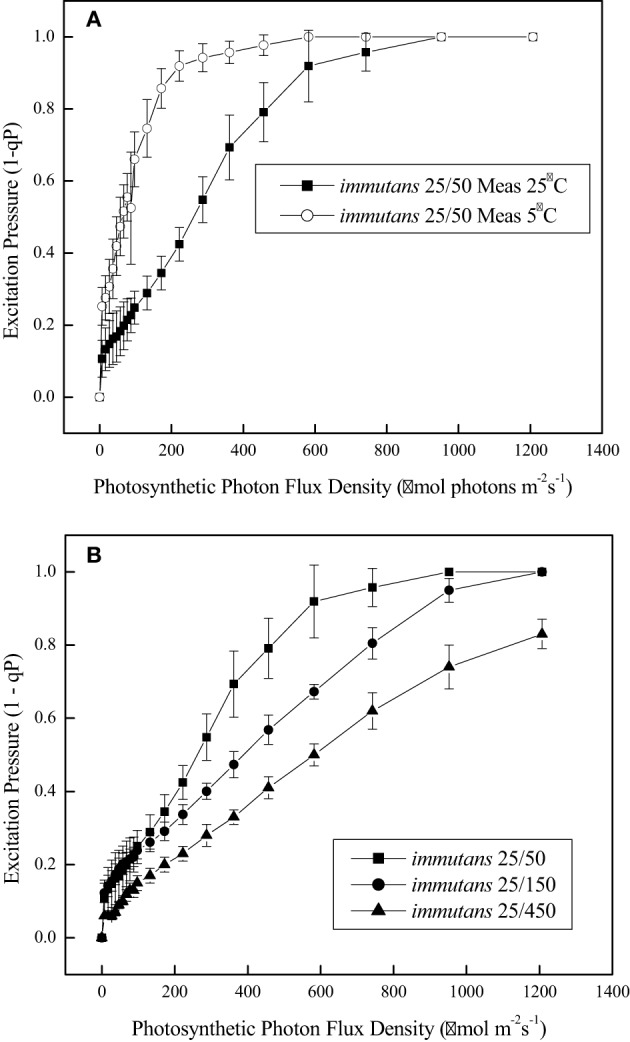
**Excitation pressure light response curves for *Arabidopsis thaliana*. (A)** Arabidopsis plants were grown at 25°C and an irradiance of 150 μmol photons m^−2^ s^−1^ and an 8 h/16 h light/dark photoperiod. Light response curves for excitation pressure were measured either at 25°C (closed symbols) or 5°C (open symbols). **(B)** Arabidopsis were exposed to growth and development at 25°C but at an irradiance of either 50 (closed squares), 150 (closed circles), or 400 μmol photons m^−2^ s^−1^ (closed diamonds) and a photoperiod of 8 h/16 h light/dark. All data are taken from Rosso et al. (2009) with permission.

**Figure 4:**
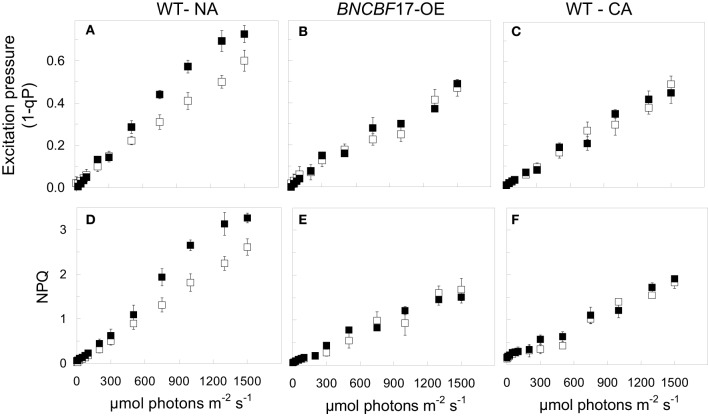
**The effects of cold acclimation and [CO_2_] on the light response curves for excitation pressure and NPQ in *Brassica napus* L. cv Westar**. **(A,D)** NA Westar WT grown at 20°C and an irradiance of 250 μmol photons m^−2^ s^−1^ with a 16 h/8 h light/dark photoperiod at either ambient (380 ppm; open symbols) or elevated CO_2_ (700 ppm CO_2_; closed symbols). **(B,E)** *BnCBF*17 overexpressor (*BnCBF*17-OE) grown at 20°C and an irradiance of 250 μmol photons m^−2^ s^−1^ with a 16 h/8 h light/dark photoperiod at either ambient (380 ppm; open symbols) or elevated CO_2_ (700 ppm; closed symbols). **(C,F)** CA Westar WT grown at 5°C and an irradiance of 250 μmol photons m^−2^ s^−1^ with a 16 h/8 h light/dark photoperiod at either ambient (380 ppm; open symbols) or elevated CO_2_ (700 ppm; closed symbols). All data are taken from Dahal et al. (2012a) with permission.

It is estimated that under optimal growth conditions only about 4.6% of the initial energy that impinges the leaf surface is conserved as fixed carbon and plant biomass (Zhu et al., 2010; Dahal et al., 2013a). A major challenge in photosynthesis research remains the enhancement in the conversion of absorbed light energy into plant biomass (Murchie et al., 2009; Zhu et al., 2010). Much recent research addressing this issue has focused primarily on targeted genetic approaches either to alter the structure and composition of the photosynthetic photosystems and their associated antenna complexes, to alter the structure of the CO_2_ fixing enzyme, Rubisco, in order to reduce the rates of photorespiration in C3 crop plants (Spreitzer and Salvucci, 2002; Long et al., 2006; Zhu et al., 2010) or to increase the potential for C4 photosynthesis in C3 plants such as rice (Edwards et al., 2008). In contrast to these targeted approaches, we propose that exploitation of the system-wide adjustments in photosynthetic performance orchestrated by cold acclimation provides a novel approach to enhance plant biomass production by co-ordinating source-sink demand which concomitantly minimizes energy loss through NPQ. To assess relative dependence on NPQ, one may compare the balance between the efficiency for light utilization for CO_2_ assimilation with the efficiency for energy dissipation through NPQ (Dahal et al., 2012a). The former can be measured as the apparent number of photons required to close 50% of PSII reaction centers (Figure 3), and the latter as the apparent number of photons required to induce one unit of NPQ (Dahal et al., 2012a). An increased capacity for the utilization of photosynthetic reductants to assimilate CO_2_, will be reflected in an increase in the quantum requirement, that is, a decrease in the initial slope of the light response curve for excitation pressure. For example, Figure 4C (open symbols) illustrates that CA *Brassica napus* WT exhibits a lower initial slope than that observed for NA *Brassica napus* WT (Figure 4A, open symbols). This indicates that cold acclimation increases the number photons required to close a PSII reaction centers by 30–50% compared to NA Brassica. Concomitantly, this is associated with a 30–50% increase in the number of photons required to induce photoprotection through NPQ in CA (Figure 4F) vs. NA *Brassica napus* WT (Figure 4D). Thus, CA *Brassica napus* exhibits a lower dependence on NPQ for photoprotection than NA *Brassica napus* due to increased rates of photosynthetic ETR and photosynthetic CO_2_ assimilation (Dahal et al., 2012a). Similar results have been reported for CA vs. NA winter rye and winter wheat which translates into a 60% increase in seed yield per plant (Dahal et al., 2013b). Thus, cold acclimation of winter rye, winter wheat, and *Brassica napus* establishes a new homeostatic state which is characterized by an increased photosynthetic capacity for CO_2_ assimilation and its conversion into biomass or energy per unit plant volume and seed production in wheat under suboptimal growth conditions (Dahal et al., 2013a,b).

In addition to enhanced photosynthetic performance and superior resistance to photoinhibition (Powles, 1984; Long et al., 1994; Edelman and Mattoo, 2008), cold acclimation suppresses stomatal conductance and transpiration rates by 30–40% regardless of the measuring temperature. The suppression of stomatal conductance observed upon cold acclimation is due, at least in part, to a decrease in leaf stomatal density. (Dahal et al., 2012a,b, 2013b). Consequently, CA plants also exhibit approximately a 3-fold increase in leaf water use efficiency (WUE) relative to NA controls (Dahal et al., 2012a,b, 2013b). However, the increase in WUE appears primarily due to a combination of a decrease in stomatal density combined with the observed increase in light saturated rates of photosynthesis.

## Enhanced photosynthetic performance is maintained during long-term growth at elevated CO_2_

It has been established that a short-term shift of C_3_ species from ambient (380 μmol C mol^−1^) to elevated CO_2_ (700 μmol C mol^−1^) results in an increase in the rates of CO_2_ assimilation (Long et al., 2004; Ainsworth and Rogers, 2007; Dahal et al., 2012c). This stimulation of photosynthesis in C_3_ plants due to elevated CO_2_ occurs because Rubisco is CO_2_ substrate-limited at ambient CO_2_ (Long et al., 2004; Tcherkez et al., 2006) and photorespiration is suppressed since CO_2_ is a competitive inhibitor of the oxygenation of RuBP by Rubisco (Long et al., 2004).

**Figure 5:**
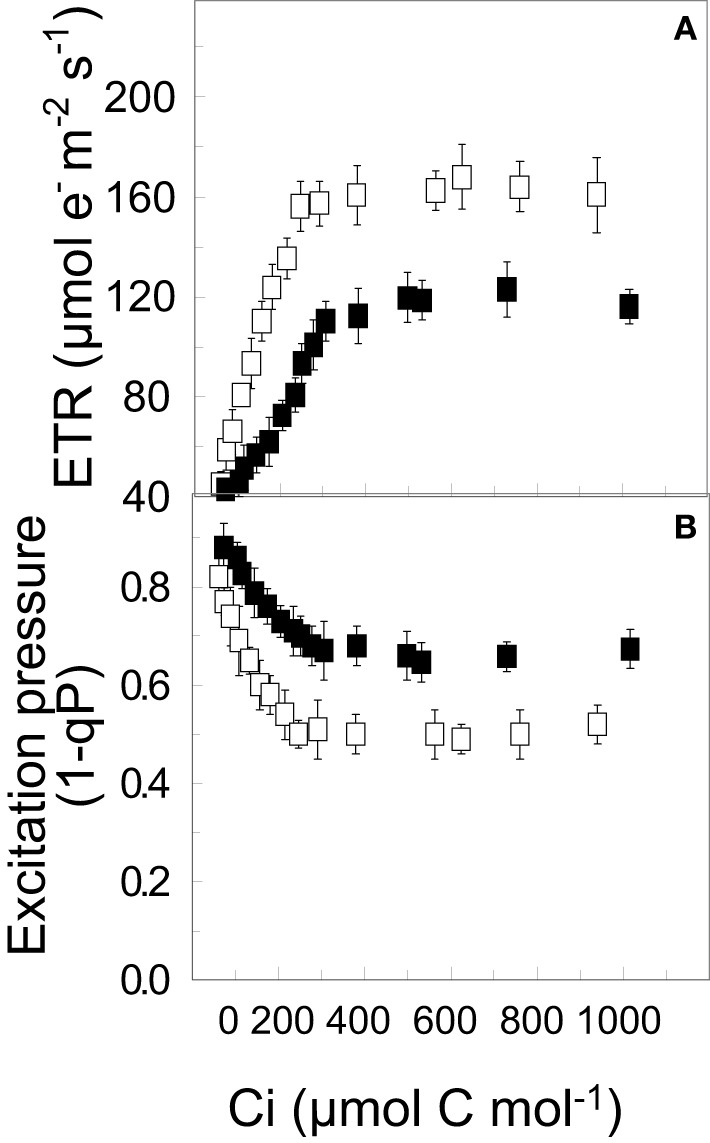
**The effects long-term growth [CO_2_] on the CO_2_ response curves for ETR (A) and excitation pressure (B) for WT *Brassica napus* L. cv Westar**. Plants were grown at 20°C and an irradiance of 250 μmol photons m^−2^ s^−1^ with a 16 h/8 h light/dark photoperiod at either ambient (380 ppm; open symbols) or elevated CO_2_ (700 ppm CO_2_; closed symbols). All data are taken from Dahal et al. (2012a) with permission.

In contrast to a short-term shift, long-term growth and development of C3 plants at high CO_2_ may lead to end product inhibition of photosynthesis due to the accumulation of sucrose in the cytosol (Stitt and Quick, 1989; Foyer et al., 1990). This feedback inhibition of photosynthetic capacity in response to growth and development at elevated CO_2_ levels may result from the combination of chloroplast Pi-limitations and the down regulation of the expression and activities of key regulatory photosynthetic enzymes (Sharkey and Vanderveer, 1989; Stitt and Quick, 1989; Drake et al., 1997; Long et al., 2004). The results in Figure 5A illustrate the inhibition light saturated rates of CO_2_ assimilation in WT *Brassica napus* grown at 25°C at either ambient (open symbols) or elevated CO_2_ (closed symbols). Consequently, WT *Brassica napus* grown at elevated CO_2_ exhibits higher excitation pressure under CO_2_ saturation conditions (Figure 5B, closed symbols) than WT *Brassica napus* grown at ambient CO_2_ conditions (Figure 5B, open symbols). However, CA *Brassica napus* WT does not suffer from feedback limited photosynthesis as a consequence of growth and development at elevated CO_2_ (Dahal et al., 2012a).

Similar to CA *Brassica napus* WT, CA winter cereals exhibit a 30–40% increase in light and CO_2_-saturated rates of photosynthesis at both ambient and elevated CO_2_. This was accompanied by a 35–50% decrease in excitation pressure and non-photochemical energy dissipation. Concomitantly, biomass increased by 28–46% and grain yield per plant by 60% (Dahal et al., 2013b). Thus, CA *Brassica napus*, winter rye, and wheat are able to maintain their superior photosynthetic performance with respect to light and CO_2_ saturated rates of CO_2_ assimilation relative to NA controls and do not exhibit feedback inhibition of photosynthesis when exposed to growth and development at elevated CO_2_. However, since this is not the case for spring rye and spring wheat cultivars, the potential for enhancement of photosynthetic performance and grain yield of cereals during long-term growth at elevated CO_2_ appears to be cultivar dependent (Dahal et al., 2013b).

Several reports have shown that a previous exposure to one type of abiotic stress can lead to enhanced tolerance to other abiotic and biotic stresses. Cold acclimation of overwintering cereals results in increased systemic resistance to plant infection by psychrophilic fungi (Hiilovaara-Teijo et al., 1999; Griffith and Yaish, 2004). Changes in temperature and CO_2_ levels affect the interactions between plant and insect pollinators as well as plant and insect herbivores due to alterations in plant phenology (DeLucia et al., 2012). Kane et al. (2013) compared the global gene expression of NA and CA Norstar winter wheat grown at either ambient or elevated CO_2_ conditions. Global gene expression analyses using microarrays combined with bioinformatics indicated that gene expression in general was more sensitive to elevated CO_2_ in NA, with 1022 genes in total, in contrast to 372 genes differentially regulated by CO_2_ in CA Norstar (Kane et al., 2013). However, the most striking effect of long-term growth at high CO_2_ was the fact that 121 of the 768 genes down-regulated by growth at elevated CO_2_ in NA Norstar are either pathogen-related or associated with systemic acquired resistance which demonstrates a major negative impact on pathogen and biotic stress defense mechanisms in NA winter wheat upon growth at high CO_2_. In contrast, CA Norstar exhibits minimal down regulation of the same genes associated with pathogen resistance and biotic stress defense. In fact, growth of CA Norstar at elevated CO_2_ caused an induction of genes involved in the biosynthesis of phytoalexins and anthocyanins as well as an up-regulation of chalcone synthase and dihydroflavonol-4-reductase (Kane et al., 2013). In addition, genes involved in photoprotection (ELIPs) and the maintenance of chloroplast stability (chaperonins) were significantly up-regulated during growth and development of CA Norstar at elevated CO_2_ (Kane et al., 2013). This is consistent with the physiological data which indicate that cold acclimation generally enhances photoprotection and the stability of the photosynthetic apparatus even under elevated CO_2_ conditions. Thus, based on the global microarray and bioinformatics analyses (Kane et al., 2013), we predict that cold acclimation not only enhances photosynthetic performance, it should also enhance inherent resistance to biotic stress. However, the latter must be confirmed experimentally and is most likely cultivar dependent.

## Overexpression of CBFs circumvents the requirement for cold acclimation

The expression of *CBFs* (C-repeat binding factors) initiate the expression of *COR* genes necessary to acquire freezing tolerance and induce a dwarf phenotype that is comparable to CA plants (Figure 2) (Jaglo-Ottosen et al., 1998; Medina et al., 1999; Zarka et al., 2003; Benedict et al., 2006; Chinnusamy et al., 2007; Thomashow, 2010; Lee and Thomashow, 2012). However, in addition to mimicking the freezing tolerance response, growth of *CBF* overexpressors at 25°C also mimicks the enhanced photosynthetic capacity (Figures 4B,E), increased plant biomass and WUE (Table 1) typically associated with cold acclimation. This is consistent with the stimulation of the rates of photosynthetic ETR and CO_2_ assimilation reported for the *BnCBF*17 overexpressor for *Brassica napus* (Dahal et al., 2012a). Furthermore, similar to CA *Brassica napus* WT, the *BnCBF*17 overexpressor also does not exhibit feedback inhibition of photosynthesis during long-term growth at elevated CO_2_ (Dahal et al., 2012a). Thus, both cold acclimation and *BnCBF*17 overexpression overcome the feedback-limited photosynthesis observed in NA control WT Brassica plants. Thus, we conclude that overexpression of *BnCBF*17 circumvents the requirement for cold acclimation to co-ordinate the system-wide adjustments in plant phenotype, photosynthetic performance and source-sink relationships in *Brassica napus*. Although *CBF* overexpression clearly alters the physiology of the shoot, little or no information is available with respect to the impact of *CBF* overexpression on root morphology, physiology, and nutrient uptake.

CA winter rye not only exhibits significantly higher rates of CO_2_ assimilation (Figure 1B, closed symbols) and ETR (Figure 1C, closed symbols) but also a lower temperature senstivity at all temperatures between 5 and 25°C than NA winter rye (Figure 1, open symbols). Similar trends were observed for NA and CA *Brassica napus* WT (Dahal et al., 2012b). However, most surprising is the fact that the temperature response curves for CO_2_ assimilation and photosynthetic ETR for CA *Brassica napus* are mimicked by simply overexpressing *BnCBF*17 even when these plants are grown at 25°C (Dahal et al., 2012b). To our knowledge, this represents the first report of such a novel manipulation of the temperature sensitivity of photosynthesis.

However, in contrast to overexpression of *BnCBF*17, overexpression of *BnCBF*5 resulted in minimal changes in photosynthetic performance *Brassica napus* (Savitch et al., 2005). Clearly, the enhanced photosynthetic performance induced by *CBF* overexpression appears to be dependent upon the specific member of this family of transcription factors that is overexpressed. Thus, a more detailed assessment of the effects of overexpression of each member of the *CBF* family of transcription factors is required.

## The potential role of CBFs

A major focus of research which attempts to elucidate the molecular mechanisms underlying plant cold acclimation and freezing tolerance has been on changes in cell membrane structure as reflected in changes in membrane lipid and fatty acid content and composition. An inherent assumption is that the plant cell membrane is the primary site that determines the potential of plants to acclimate to low temperature and consequently exhibit maximum freezing tolerance (Levitt, 1980; Steponkus, 1984; Guy, 1990; Murata and Los, 1997; Thomashow, 2001; Los and Murata, 2002; Chinnusamy et al., 2007). In cyanobacteria, two-component histidine kinases activated by changes in membrane viscosity have been shown to stimulate the expression of specific fatty acid desaturases. This results in the modulation of membrane fluidity in response to low temperature stress in cyanobacteria (Los and Murata, 2002; Xin, 2002; Los et al., 2013). Low temperature-induced alterations in the physical structure of plant cell membranes also activates Ca^2+^ channels and rapidly generates a Ca^2+^signal (Monroy et al., 1998; Plieth et al., 1999; Orvar et al., 2000). The rapid increase in cytosolic Ca^2+^ activates a cytosolic protein kinase whose substrate is ICE1. The phosphorylation of ICE1 is required for the induction of a family *CBF* transcription factors which regulate the expression of *COR* genes essential for acquisition of maximal freezing tolerance (Jaglo-Ottosen et al., 1998; Medina et al., 1999; Zarka et al., 2003; Benedict et al., 2006; Chinnusamy et al., 2007; Thomashow, 2010; Lee and Thomashow, 2012). The expression of *CBF3* in *Arabidopsis thaliana* is positively regulated by the constitutively expressed ICE1 gene, the product of which binds to multiple regulatory elements present in the *CBF3* promoter and stimulates its transcription (Chinnusamy et al., 2007; Thomashow, 2010). The action of ICE1, in turn, is tightly regulated by SIZ1, a SUMO E3 ligase, and HOS1 which is a RING finger E3 ligase (Lee et al., 2001; Zhu et al., 2004; Dong et al., 2006; Miura et al., 2007; Hua, 2009; Miura and Ohta, 2010; Thomashow, 2010).

Enhanced freezing tolerance has been reported in plants such as *A. thaliana*, canola (*Brassica napus* L.), tomato (*Solanum lycopersicum* L.), and poplar (*Populus balsamifera* subsp. *trichocarpa*) in which *CBF*s have been over-expressed (Jaglo-Ottosen et al., 1998; Hsieh et al., 2002; Savitch et al., 2005; Benedict et al., 2006). In herbaceous plants, *CBF* overexpression is typically associated with the dwarf phenotype observed during cold acclimation even though the plant has been grown at warm temperatures (Hsieh et al., 2002; Savitch et al., 2005; Achard et al., 2008; Huang et al., 2009; Dahal et al., 2013a). Thus, the *CBF* regulon not only regulates freezing tolerance but also has dramatic affects on plant development that are considered to be controlled by the family of plant phytochromes as well as phytohormones (Franklin, 2009; Kurepin et al., 2013).

All photoautotrophic organisms sense changes in light quality as a “biogenic signal” through photoreceptors such as the phytochromes and cryptochromes to regulate photomorphogenesis (Pogson et al., 2008; Sakamoto et al., 2008; Waters and Langdale, 2009; Jarvis and Lopez-Juez, 2013). However, photoautotrophs also sense changes in light intensity as fluctuations in energy input for photosynthesis as an “operational signal” to maintain a cellular energy balance that is, photostasis (Hüner et al., 2003, 2012; Pogson et al., 2008). Photoautotrophs must balance the extremely fast rates of cellular energy input through the temperature-insensitive photochemical reactions of photosystem I and photosystem II with the slower, and temperature-dependent enzyme processes involved in either energy dissipation as heat or energy utilization through primary C, N, and S assimilation (Foyer et al., 2000; Foyer and Noctor, 2002; Murchie et al., 2009; Hüner and Grodzinski, 2011; Hüner et al., 2012, 2013). The regulation of the phytochrome-dependent, photomorphogenic signal transduction pathways (Franklin and Whitelam, 2007; Pogson et al., 2008; Franklin, 2009) as well as the retrograde sensing/signaling pathways between the chloroplast and the nucleus (Koussevitzky et al., 2000; Nott et al., 2006; Lim et al., 2007; Fernandez and Strand, 2008; Pogson et al., 2008; Woodson and Chory, 2008) involved in remodeling of the photosynthetic apparatus are both light- and temperature-dependent (Li et al., 1993; Franklin, 2009; Kim and Apel, 2013; Hüner et al., 2012, 2013). Thus, cross-talk between the biogenic signaling pathways and the complex operational signaling networks must exist since both govern plant plasticity in response to an ever-changing environment. Elucidation of nature of this complex integration of light sensing/signaling networks remains a major challenge for future research.

**Figure 6:**
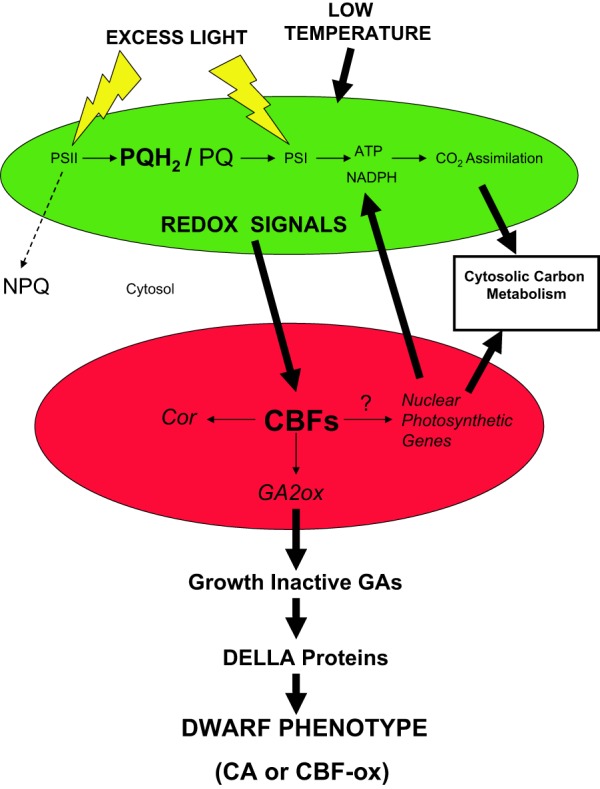
**A proposed model illustrating the role of CBFs in governing the system-wide integration of excitation pressure, photosynthetic capacity and the dwarf phenotype**. Growth of plants either under excess light or low temperature poises the intersystem photosynthetic electron transport chain of the chloroplast (green) in a reduced state as indicated by the accumulation of plastoquinol (PQH_2_) relative to plastoquinone (PQ). This is due to an imbalance between the flux of energy absorbed through extremely fast, temperature-independent photochemistry occurring within the PSII and PSI reaction centers relative to the energy utilized either through much slower, temperature-sensitive biochemical reactions involved in CO_2_ assimilation, cytosolic carbon metabolism, and export or the energy dissipated as heat through NPQ. Such an energy imbalance increases excitation pressure. Through retrograde signaling pathways, the myriad redox signals generated by the chloroplast due to the increased excitation pressure are transduced to the nucleus (red) to modulate *CBF* expression. This leads to the activation not only of *COR* genes important in freezing tolerance but also the activation of nuclear encoded photosynthetic genes associated with thylakoid membrane protein complexes involved in photosynthetic electron transport as well as enzymes of the Calvin–Benson Cycle and cytosolic sucrose biosynthesis. The latter results in the increased capacity for CO_2_ assimilation upon growth under high excitation pressure. DELLA proteins are repressors of stem elongation. Growth-active GAs stimulate stem elongation by activating the breakdown of DELLA proteins, and thus, de-repressing stem elongation. *CBFs* affect the biosynthetic pathways for GA biosynthesis by activating nuclear encoded *GA2ox* genes which predisposes plants to accumulate growth-inactive GAs (Kurepin et al., 2013). Consequently, activation of *CBFs* by excitation pressure results in the accumulation of growth inactive GAs which maintains the plant in a repressed growth state due to the accumulation of DELLA proteins. As illustrated in the model, the low growth temperature or high light requirement usually needed to elicit enhanced photosynthetic capacity and the dwarf phenotype can be circumvented by overexpression over-expression of *CBFs* (*CBF-ox*). The enhanced photosynthetic capacity induced either by excitation pressure or overexpression of CBFs, minimizes the requirement for photoprotection of the chloroplast through NPQ as illustrated by the broken arrow. The mechanism by which *CBFs* activate the expression of known nuclear photosynthetic genes is presently unknown.

Recently, we proposed that CBFs act as master regulators of cold acclimation and photosynthetic performance which integrate both the upstream and downstream signals (Kurepin et al., 2013). In our model illustrated in Figure 6, we propose that redox input signals from chloroplasts (green), manifested as modulation of excitation pressure, are transduced to the nucleus (red) via retrograde regulation and stimulate *CBF* expression. Recently, we reported that *CBF3* in *Arabidopsis thaliana* is regulated by excitation pressure rather than low temperature *per se* (Bode, 2012; Kurepin et al., 2013). Furthermore, we suggest that the phenotype and physiological properties of CA plants occurs by the stimulation of *CBF* gene expression to induce not only freezing tolerance but also genes associated with photosynthesis, cytosolic carbon metabolism and respiration in addition to the activation of *GA2ox* genes which decreases the levels of growth-active GAs. Growth-active GAs normally bind to DELLA proteins to de-repress genes involved in cell growth and stem elongation (Peng and Harberd, 1997; Peng et al., 1997, 1999). Thus, accumulation of growth-inactive GAs maintains levels of DELLA proteins such growth and stem elongation are repressed which generates a dwarf phenotype. This dwarf phenotype exhibits enhanced photosynthetic performance and increased dry biomass per unit plant volume (Savitch et al., 2005; Dahal et al., 2012a, 2013a,b) coupled with enhanced seed yield in CA wheat (Dahal et al., 2013b). We conclude that plants sense low temperature through a complex sensing/signaling network which integrates changes in temperature and actinic light intensity through modulation of operational signals emanating from the chloroplast due to chloroplast redox imbalance or excitation pressure (Hüner et al., 1998; Ensminger et al., 2006; Hüner et al., 2012, 2013; Kurepin et al., 2013). Thus, the chloroplast should not only be considered the primary energy in transformer but also a major cellular energy sensor for detecting changes in the environment (Hüner et al., 1998; Pfannschmidt, 2003; Murchie et al., 2009). However, operational chloroplast redox signaling must be integrated with regulation associated changes in light quality sensed through photoreceptors such as phytochrome (Franklin and Whitelam, 2007; Franklin, 2009) as well as through specific cell membrane, low temperature sensors (Murata and Los, 1997; Plieth et al., 1999; Knight and Knight, 2000; Los and Murata, 2002; Los et al., 2013) in order to establish a new CA, homeostatic state.

## CBFs, climate change, and crop productivity

The intergovernmental panel on climate change has predicted that the atmospheric CO_2_ concentration will double from present 380 μmol C mol^−1^ to ca. 700 μmol C mol^−1^ by the end of the twenty-first century which may be coupled to an increase in average global temperature (IPCC, 2007). The predicted increase in atmospheric CO_2_ and drastic temperature changes associated with global climate change may increase the severity of water stress as well as the incidence of biotic stresses (Hatfield et al., 2011; DeLucia et al., 2012; Vandegeer et al., 2012). Such sub-optimal growth conditions due to climate change may substantially affect the photosynthetic performance of plants and hence, plant biomass production and crop seed yield. To add to the complexity of the effects of climate change on crop productivity, the sub-optimal growth conditions associated with climate change is occurring at a time when the world population is likely to increase by about 30% by 2050 (UN Report, 2011). It has been predicted that the world food demand will double in the next 20 years due to increased world population as well as increased per capita food consumption (Murchie et al., 2009). This has triggered an immediate need to substantially enhance seed yield and productivity of major food crops such as rice, wheat, and maize to meet the nutritional demand of the increasing population. This urgent need is exacerbated by the fact that the projected increased food demand is occurring at a time when continued losses of prime agricultural land is occurring due to urbanization, desertification in most of the developing countries, an increasing proportion of grain is being diverted for animal feed and biofuel generation which are combined with the suboptimal growth conditions for crop production due to climate change (Murchie et al., 2009; Zhu et al., 2010).

During the 1960s and 1970s wheat yields worldwide increased significantly due to the development of new varieties and increased availability and use of N fertilizers. This led to the “green revolution” which was characterized by short, sturdy, semi-dwarf genotypes that exhibit an increased HI and a decreased tendency to lodge (Peng et al., 1999). Subsequent molecular and genetic analyses of these semi-dwarf varieties showed that this phenotype was conferred by dominant mutant dwarfing alleles which are orthologs of the *Gibberellic Acid Insensitive* gene (*GAI*) of *Arabidopsis thaliana* (Peng et al., 1999). In wild type, GAI represses stem elongation at low endogenous GA levels but under high endogenous of GA levels, GA binds to GAI and de-represses the inhibitory effect of GAI which results in normal stem elongation and an elongated phenotype (Peng et al., 1997, 1999; Peng and Harberd, 1997). In the dwarf plants, the mutant gene, *gai*, is present which does not bind endogenous GA. Consequently, mutants retain a dwarf phenotype irrespective of endogenous GA levels.

Since growth active GAs stimulate stem elongation (Hopkins and Hüner, 2008), application of exogenous GAs to *CBF*-overexpressing plants rescues the dwarf phenotype. In contrast, application of other plant hormones known to affect shoot growth does not rescue the dwarf phenotype (Hsieh et al., 2002; Achard et al., 2008). Published reports indicate that overexpression of *CBF*s results in increased levels of DELLA proteins (Achard et al., 2008; Kurepin et al., 2013) which repress stem elongation and leads to a dwarf phenotype (Peng and Harberd, 1997; Peng et al., 1997). Recent evidence indicates that excitation pressure not only regulates the expression of photosynthetic genes (Hüner et al., 1998; Rosso et al., 2009) but also activates *AtCBF*3 expression in *Arabidopsis thaliana* (Bode, 2012; Kurepin et al., 2013). Since growth- active GAs stimulate the degradation of DELLA proteins (Achard et al., 2008), the accumulation of DELLA proteins, in part, reflect the increased expression of *GA2ox* genes which leads to the accumulation of growth-inactive GAs (Kurepin et al., 2013). Thus, the published evidence is consistent with the notion that excitation pressure governs the dwarf phenotype through the activation *CBF* gene expression which, in turn, may control the expression of G*A2ox* genes which consequently reduces the accumulation of growth active GAs in CA plants as well as plants grown under excessive irradiance (Gray et al., 1997; Achard et al., 2008; Kurepin et al., 2013). Consequently, plants grown under either excessive irradiance or low temperature exhibit a dwarf phenotype which is mimicked by overexpression of *CBF*s (Gilmour et al., 2000; Savitch et al., 2005; Dahal et al., 2012a) (Figure 6).

The model illustrated in Figure 6 is consistent with the thesis that photosynthesis and the chloroplast have a dual role—not only do they represent the major energy transformers of sunlight into biomass, they also govern a broad range of physiological and plant developmental processes which have a direct impact on phenotypic plasticity. This model is consistent with the “grand design of photosynthesis” first proposed by Arnon (1982) and subsequently supported by a series of reviews in the last 20 years focused on photoacclimation (Anderson et al., 1995), acclimation to low temperature and light (Hüner et al., 1998; Pfannschmidt, 2003), acclimation and cell death (Mullineaux and Baker, 2010) as well as plant growth and productivity (Murchie et al., 2009; Kurepin et al., 2013).

The maintenance of crop yield stability through enhanced tolerance to environmental stresses such as drought, low and high temperature, as well as biotic stress associated with global climate change remains a crucial challenge to maximize future crop productivity worldwide (Powell et al., 2012). We suggest that targeting the *CBF* family of transcription factors in major crop species may be a novel approach to improve crop productivity through increased photosynthetic performance coupled with increased WUE and the potential for enhanced resistance to biotic stress. However, an important caveat to this approach is that low temperature induction of *CBF*s in *Arabidopsis thaliana* also activates the expression of Flowering Locus C (*FLC*), a negative regulator of flowering (Seo et al., 2009). This results in a significant delay in flowering time which reflects an evolutionary mechanism to prevent premature floral development during the late fall or early spring seasons. Similarly, overexpression of *AtCBF*3 delayed the onset of bolting by 4 to 9 days at 20°C in Arabidopsis (Gilmour et al., 2000). Such a delay in flowering time has been confirmed in subsequent studies (Magome et al., 2004; Seo et al., 2009). Furthermore, seed yield also appears to be reduced in CBF overexpressors compared to wild type when grown at warm temperatures (Liu et al., 1998; Gilmour et al., 2000). Thus, when grown under optimal conditions, the increased photosynthetic performance induced by *CBF* overexpression may not translate into increased seed yield. However, it is crucial to compare the conversion of plant biomass into seed yield in *CBF* overexpressors with wild type plants when grown under suboptimal growth conditions. The prediction is that the *CBF* overexpressor should out perform the wild type under such conditions. Further research is required to confirm this prediction.

### Conflict of interest statement

The authors declare that the research was conducted in the absence of any commercial or financial relationships that could be construed as a potential conflict of interest.
